# Personalized chemotherapy guidance using patient-derived scaffolds from peritoneal colorectal metastases

**DOI:** 10.1186/s40164-026-00801-4

**Published:** 2026-07-01

**Authors:** Simona Salerno, André Holdfeldt, Anders Ståhlberg, Marianne Quiding Järbrink, Göran Landberg, Elinor Bexe Lindskog

**Affiliations:** 1 Department of Laboratory Medicine, Sahlgrenska Center for Cancer Research, Institute of Biomedicine, Sahlgrenska Academy at the University of Gothenburg, Gothenburg, Sweden; 2 Wallenberg Centre for Molecular and Translational Medicine, University of Gothenburg, Gothenburg, Sweden; 3 Department of Clinical Genetics and Genomics, Region Västra Götaland, Sahlgrenska University Hospital, Gothenburg, Sweden; 4 Science for Life Laboratory, Institute of Biomedicine, University of Gothenburg, Gothenburg, Sweden; 5 Department of Pathology, Region Västra Götaland, Sahlgrenska University Hospital, Gothenburg, Sweden; 6 Department of Surgery, Region Västra Götaland, Sahlgrenska University Hospital, Gothenburg, Sweden; 7 Surgical Oncology Laboratory, Department of Surgery, Institute of Clinical Sciences, Sahlgrenska Academy at the University of Gothenburg, Gothenburg, Sweden; 8 Department of Microbiology and Immunology, Institute of Biomedicine, Gothenburg, Sweden

**Keywords:** Peritoneal metastases, Colorectal cancer, Patient-derived scaffolds, HIPEC, Chemotherapy

## Abstract

Peritoneal metastases from colorectal cancer are associated with poor survival and limited therapeutic efficacy. To model patient-specific treatment responses, we generated patient-derived scaffolds (PDS) through tumor decellularization, preserving critical microenvironmental features for functional ex vivo analysis. PDS from 75 metastases were recellularized with HT29 cells and treated with 5-fluorouracil (5-FU), mitomycin C, or oxaliplatin. Gene expression related to proliferation, epithelial phenotype, epithelial-mesenchymal transition, and stemness was quantified and integrated into a composite treatment score to estimate the most favorable therapy for each patient. Treatment responses were influenced by both drug exposure and the patient-specific microenvironment. Importantly, subcluster distributions differed across treatment arms, supporting the concept that therapeutic response is modulated by microenvironmental context rather than being solely drug dependent. These findings were supported in a smaller validation cohort using HCT116 and DLD-1 cells recellularized into selected PDS. Treatment scoring based on HT29 cells revealed heterogeneous sensitivity patterns, with 38.7% of PDS responding best to 5-FU, 36% to mitomycin C, and 25.3% to oxaliplatin. Patients with more than two favorable treatment scores had improved disease-free survival (*p* = 0.0415). The PDS platform enables functional profiling of chemotherapy-response and may support individualized treatment strategies for colorectal cancer patients with peritoneal metastases.

**Supplementary Information:**

The online version contains supplementary material available at 10.1186/s40164-026-00801-4.

**To the Editor**,

Peritoneal carcinomatosis in colorectal cancer is associated with poor prognosis and has traditionally been viewed as a palliative condition [1, 2]. Although cytoreductive surgery combined with hyperthermic intraperitoneal chemotherapy (HIPEC) offers a treatment option for selected patients, its clinical benefit remains uncertain due to inconsistent trial outcomes [3–6]. This underscores the need for preclinical models that can capture patient-specific variability and inform personalized treatment strategies. The patient-derived scaffold (PDS) model addresses this need by providing a rapid and reproducible platform that preserves the native extracellular matrix and microenvironmental complexity through tumor tissue decellularization [7–11]. Histological characterization and proteomic analyses confirm that PDSs retain site-specific ECM components and molecular imprints of the original tumor niche [8, 12, 13]. Functional decoding of these environments can be achieved by monitoring gene expression changes in adapting cell lines, offering insights into tumor–stroma interactions and treatment responses.

In this study we generated PDSs from peritoneal metastases of 75 colorectal cancer patients. Removal of cellular material was confirmed by quantitative DNA depletion analysis (Supplementary Fig. 4B). These scaffolds were seeded with HT29 cells, which were used as a standardized reporter system to isolate microenvironment-driven effects. Seeded PDSs were exposed ex vivo to systemic chemotherapy agent 5-FU (10 mM), or HIPEC agents Mitomycin C (100 µM), or Oxaliplatin (100 µM) for 48 h. Untreated controls were included to isolate PDS-specific effects. Treatment doses were optimized to achieve ~ 50% reduction in RNA yield (Supplementary Fig. 1), and a schematic overview of the workflow is provided in Supplementary Fig. 2A. All experiments were performed under normothermic conditions (37 °C), representing a simplification of clinical HIPEC to enable controlled comparison of microenvironment-dependent transcriptional responses.

HT29 cells cultured on peritoneal PDSs adopted a transcriptional profile characterized by reduced proliferation and increased expression of EMT and stemness markers, consistent with phenotypic shifts induced by 3D culture [14]. While overall profiles were similar, subtle but consistent gene expression differences across PDSs reflected patient-specific microenvironmental imprints. PCA, tSNE, and hierarchical clustering revealed considerable heterogeneity among untreated PDSs (Fig. 1A, Supplementary Fig. 2C–E), with selected gene markers correlating with clinical features (Fig. 1B). These findings underscore the biological complexity captured by the PDS model, extending beyond conventional clinical classification.

**Fig. 1:**
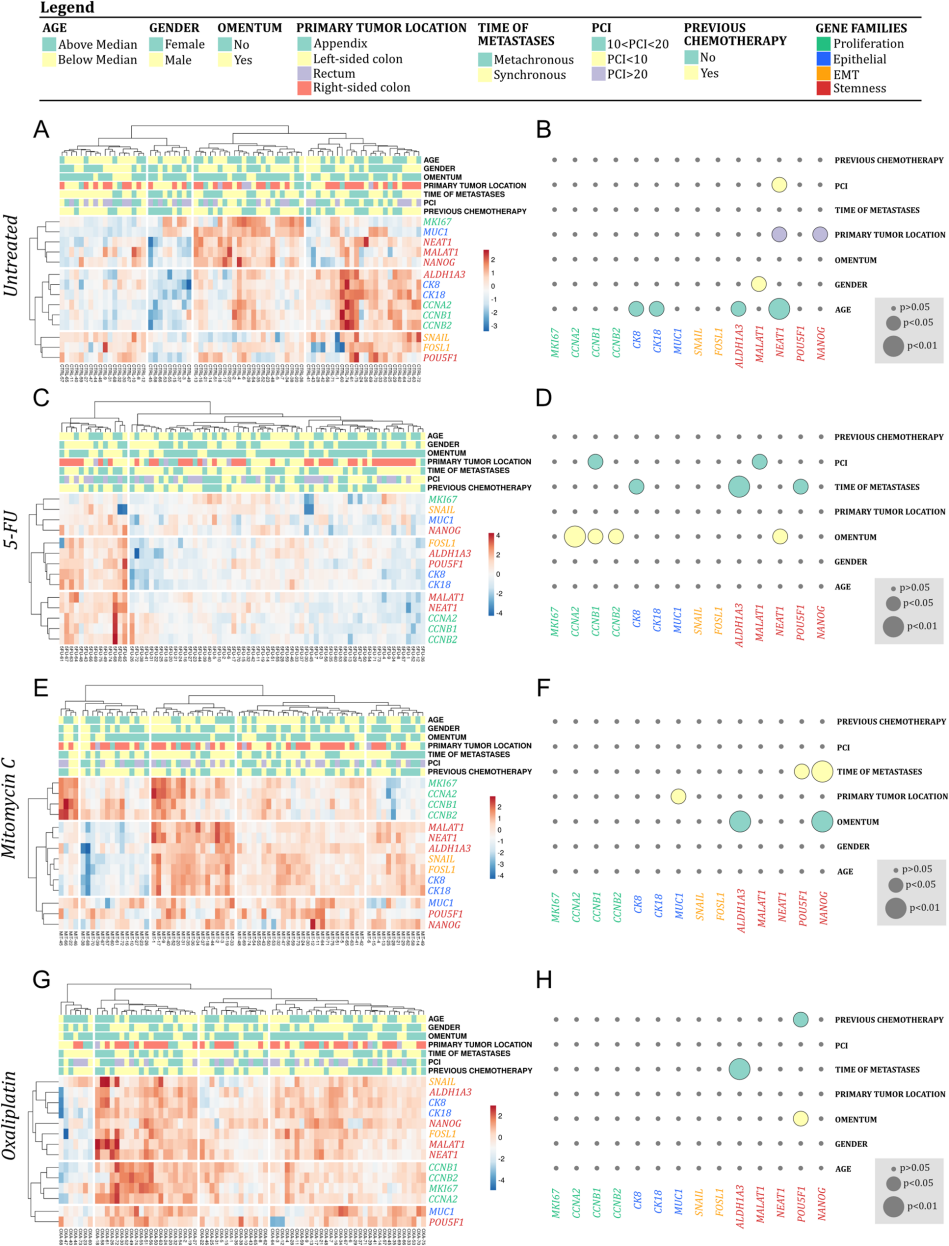
Patient-specific microenvironment influences chemotherapy responses. Unsupervised hierarchical clustering analysis of PDS-derived gene expression data under different treatment conditions: **A** untreated; **C** 5-FU-treated, **E** mitomycin C-treated and **G** oxaliplatin-treated PDS. Rows represent gene markers, columns represent individual PDS samples. Data are rows-centered with unit variance scaling applied. Both rows and columns were clustered using Euclidian distance and Ward linkage. Analysis and visualization was performed with ClustVis (https://biit.cs.ut.ee/clustvis/). Bubble plots summarizing correlations of qPCR data obtained from **B** untreated, **D** 5-FU treated, **F** mitomycin C-treated and **H** oxaliplatin-treated PDS cultures with clinical information of the corresponding patients. Each dot represents the statistical correlation between one gene and one clinical parameter. The size of the dot indicates the level of significance; the color of the dot indicated the group in which the expression level is highest. The statistical tests used are Mann Whitney U test or Kruskal Wallis test

Chemotherapy exposure demonstrated the ability of the PDS model to capture patient-specific microenvironmental responses. Following 5-FU treatment, unsupervised clustering revealed largely homogeneous gene expression profiles, with one distinct subgroup diverging across multiple markers (Fig. 1C). Gene expression correlated with clinical parameters including omental involvement, metastasis timing, and PCI score (Fig. 1D). Notably, three of four proliferation markers were elevated in omental metastases (*p* < 0.05), consistent with sustained HT29 proliferation in omentum-derived PDS. This aligns with the omentum’s vascularized, macrophage-rich niche, which may promote metastasis and chemoresistance [15, 16]. Mitomycin C–treated PDS formed five molecular subclusters with variable proliferation, EMT, and stemness profiles, again correlating with clinical features (Fig. 1E, F). Oxaliplatin-treated PDS generated four subclusters with consistent transcriptional patterns; although some clinical associations were observed, these did not reach statistical significance (Fig. 1G, H).

Distinct gene expression subclusters were identified for each chemotherapy agent (Supplementary Fig. 3A–C), with no overlap between treatments, indicating that the PDS model captures unique microenvironment-driven responses. To represent how a given microenvironment modulates transcriptional responses across different agents, we developed a treatment score based on gene expression changes in treated PDS cultures, estimating the likely efficacy of 5-FU, Mitomycin C, or Oxaliplatin for each PDS culture (Fig. 2A). A favorable response was defined as reduced expression of gene markers associated with malignant traits as proliferation, cancer stemness, EMT and epithelial markers CK8, CK18, and MUC1, all linked to poor clinical outcomes [17–19]. Based on this scoring, 38.7% of patients were predicted to respond best to 5-FU, 36% to Mitomycin C, and 25.3% to Oxaliplatin (Fig. 2B), underscoring the absence of a universal optimal therapy and highlighting the influence of the metastatic niche on drug response. Kaplan-Meier analysis revealed early separation between patients with multiple positive treatment scores and those with fewer, with statistical significance in the Gehan-Breslow-Wilcoxon test (*p* = 0.0415), though not in the log-rank test (*p* = 0.1065) (Fig. 2C). A similar trend was observed using median-based stratification (Supplementary Fig. 2D–E).

**Fig. 2:**
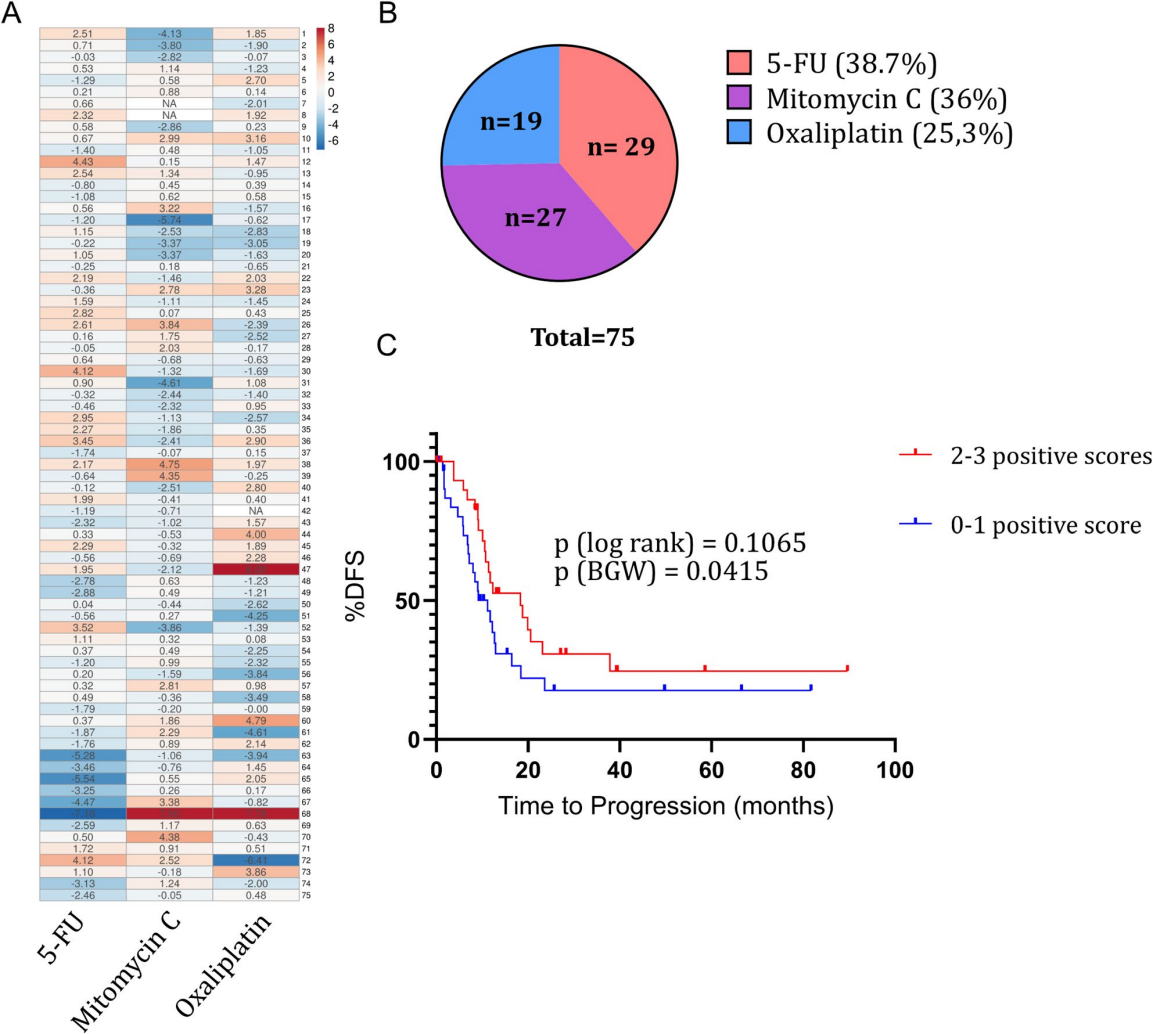
Simulating therapeutic decision making through peritoneal metastases PDS cultures. **A** Heatmap showing treatment scores across individual patients (rows) and treatments (columns), with higher values indicating greater predicted benefit. **B** Pie chart summarizing the number and proportion of patients whose top treatment to 5-FU, Mitomycin C and Oxaliplatin. **C** Kaplan–Meier analysis of Disease-free survival (DFS) stratified by treatment score profiles. Patients were categorized into 2 groups based on the number of positive treatment scores. Group with 2–3 positive scores (*n* = 31) and group with 0–1 positive scores (*n* = 32)

Despite limitations in cohort size and retrospective analyses, our findings demonstrate that the PDS model captures clinically relevant, microenvironment-specific treatment responses in colorectal cancer. In line with the primary data, differential drug responses were confirmed in a smaller validation cohort using HCT116 and DLD-1–repopulated PDS (Supplementary Fig. 4A). The gene expression-based treatment score provides a framework for identifying patient-specific therapeutic preferences, highlighting the absence of a universal optimal regimen and the influence of the metastatic niche on drug efficacy. Early survival separation suggests potential clinical relevance, although these exploratory results are hypothesis-generating. As a proof-of-concept platform, PDS enables functional interrogation of microenvironment-dependent chemotherapy responses and warrants prospective validation in larger cohorts.

## Supplementary Information

Below is the link to the electronic supplementary material.

Supplementary Material 1.

## Data Availability

Data and materials are available from the corresponding authors on reasonable request.

## Abbreviations

HIPEC: Hyperthermic intraperitoneal chemotherapy
PDS: Patient-derived scaffold
5-FU: 5-fluorouracil
