# Supplementary material for: Personalized chemotherapy guidance using patient-derived scaffolds from peritoneal colorectal metastases
Source: Exp Hematol Oncol. 2026 Jul 1;15:58. doi: 10.1186/s40164-026-00801-4 (PMC13321693; doi:10.1186/s40164-026-00801-4)
Supplement: Supplementary file 1 — Supplementary Material 1. [file 40164_2026_801_MOESM1_ESM.docx]

**SUPPLEMENTARY FIGURES**

**
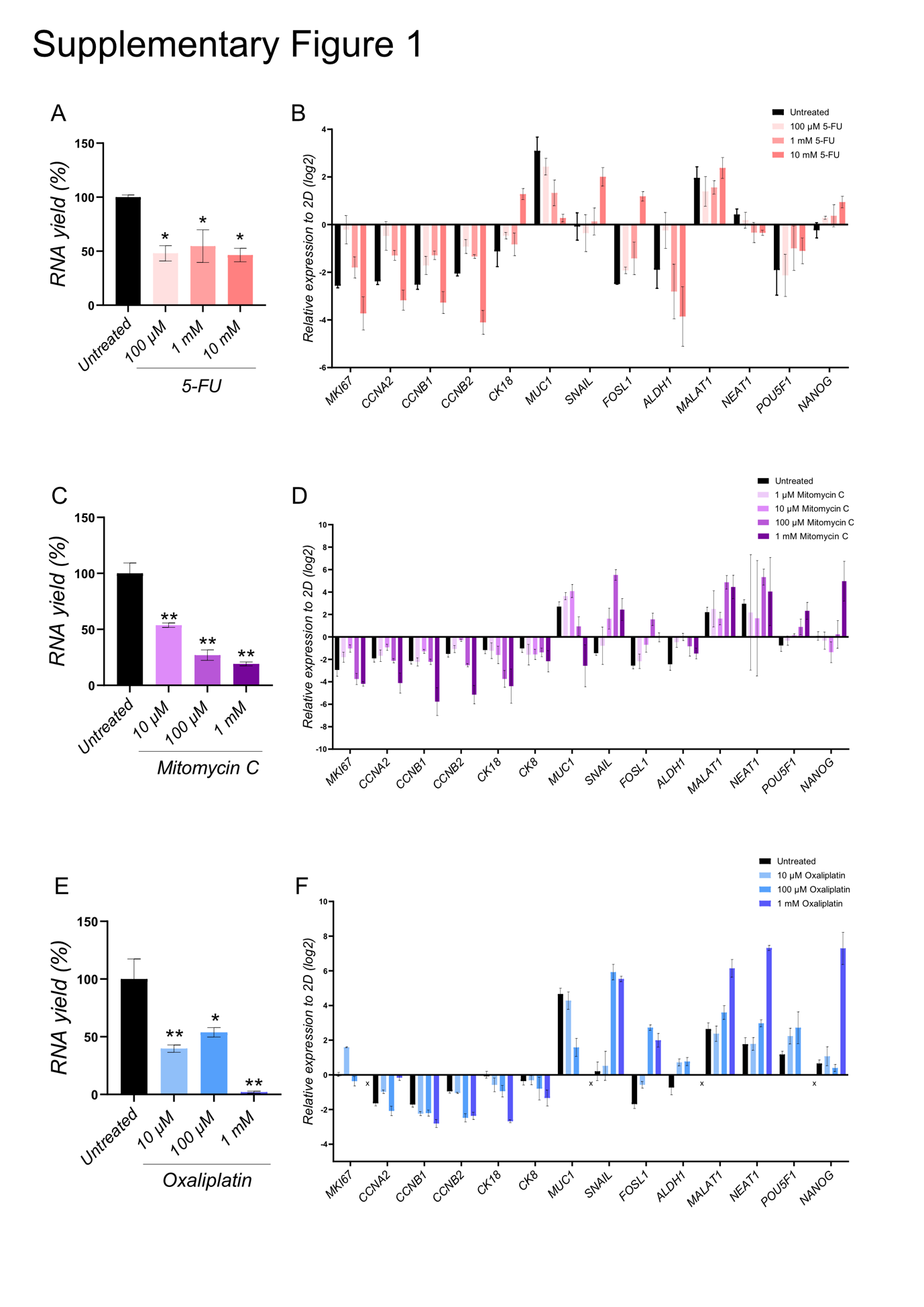
**

**Supplementary Figure 1: Chemotherapy dose optimization in PDS**

Percentage RNA yield of HT29 cells cultured in PDS following treatment with increasing concentrations of **(A)** 5-FU, **(C)** Mitomycin and **(E)** Oxaliplatin (Mean ± SEM, *p<0.05, **p<0.01, one-way ANOVA), and qPCR data showing gene expression before and after treatment with increasing concentrations of **B**. 5FU, **D**. Mitomycin and **F**. Oxaliplatin.


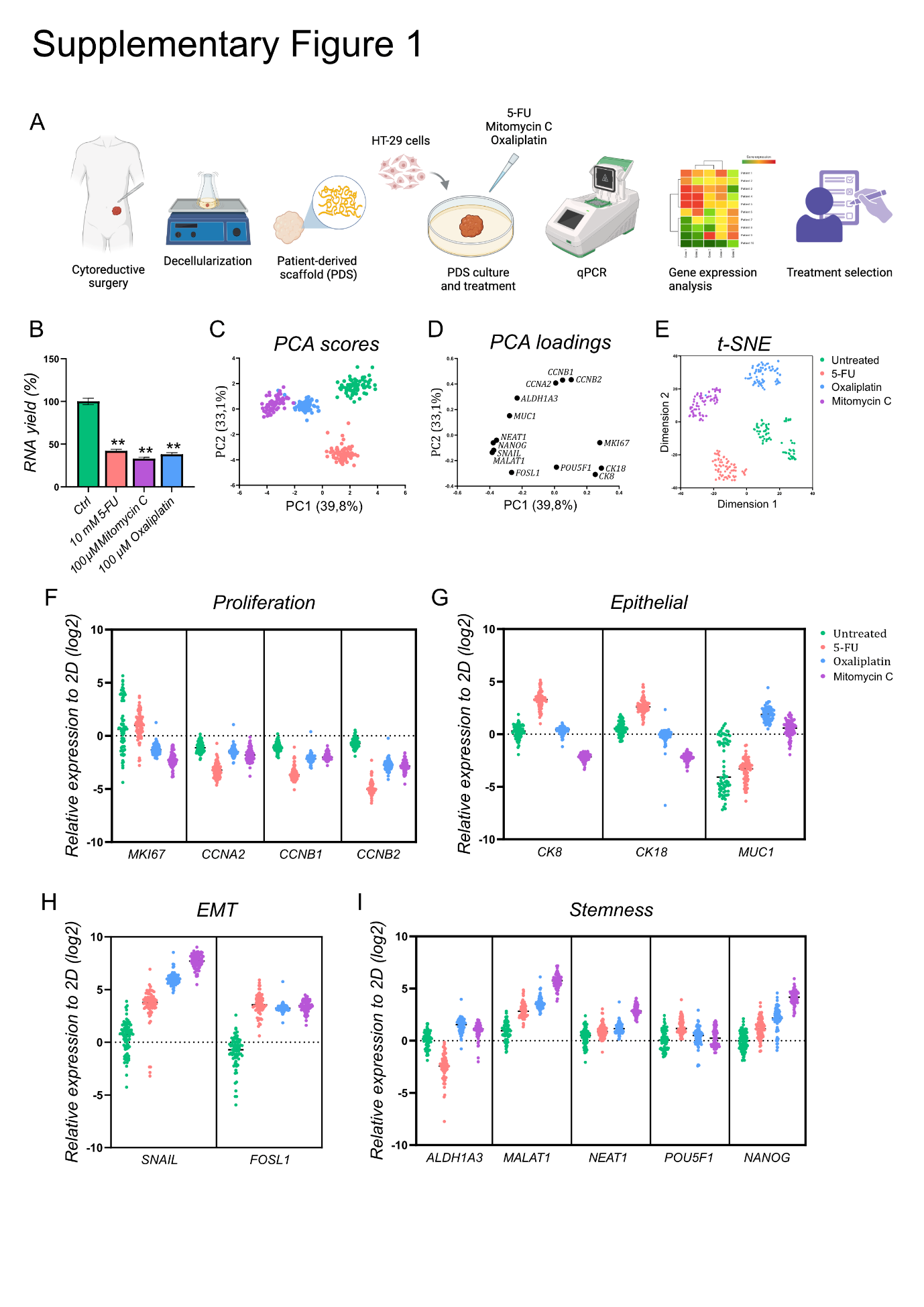


**Supplementary Figure 2. Transcriptional profiling of PDS cultures treated with chemotherapeutic agents**

**(A)** Schematic overview of experimental workflow. Created with Biorender (<https://www.biorender.com/>). **(B)** Percentage RNA yield of HT29 cells cultured in PDS following treatment with 5-FU, Oxaliplatin and Mitomycin C relative to untreated control PDS cultures. Mean ± SEM, **p<0.01, one-way ANOVA. **(C-D)** Principal component analysis of gene expression data of PDS cultures. **(E)** t-SNE analysis of gene expression data of PDS cultures. **(F-I)**. qPCR data showing gene expression before and after treatments. Dots indicate individual PDS cultures.


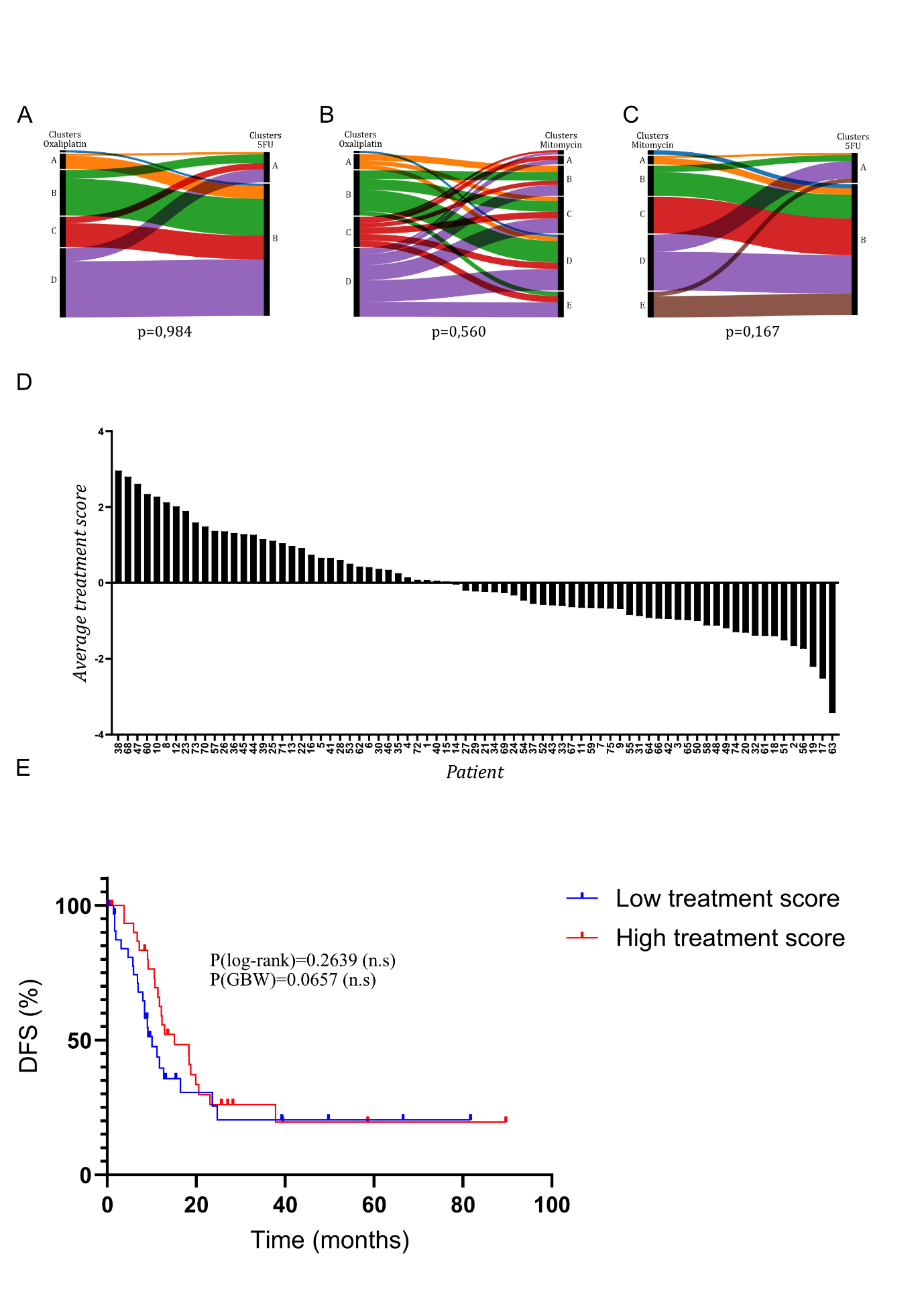


**Supplementary Figure 3: Cluster correspondence between treatment conditions**

Alluvial plots showing the correspondence of sample cluster assignments across the three treatment conditions. **(A)** Cluster correspondence between Oxaliplatin- and 5FU-treated samples; **(B)** Cluster correspondence between Oxaliplatin- and Mitomycin-treated samples; **(C)** Cluster correspondence between Mitomycin- and 5FU-treated samples. Each ribbon represents the number of samples shared between clusters under each treatment condition. Clusters are labeled according to hierarchical clustering within each treatment condition. Statistical significance was assessed using Pearson’s Chi square analysis. Plots were created using RAWGraphs 2.0 (<https://www.rawgraphs.io/>). **(D**) Waterfall plot displaying the average treatment score for each patient, ranked from highest to lowest. **(E)** Kaplan-Meier analysis of Disease free survival (DFS) stratified by average treatment score. Patients were categorized into two groups using the median average treatment score as cutoff.


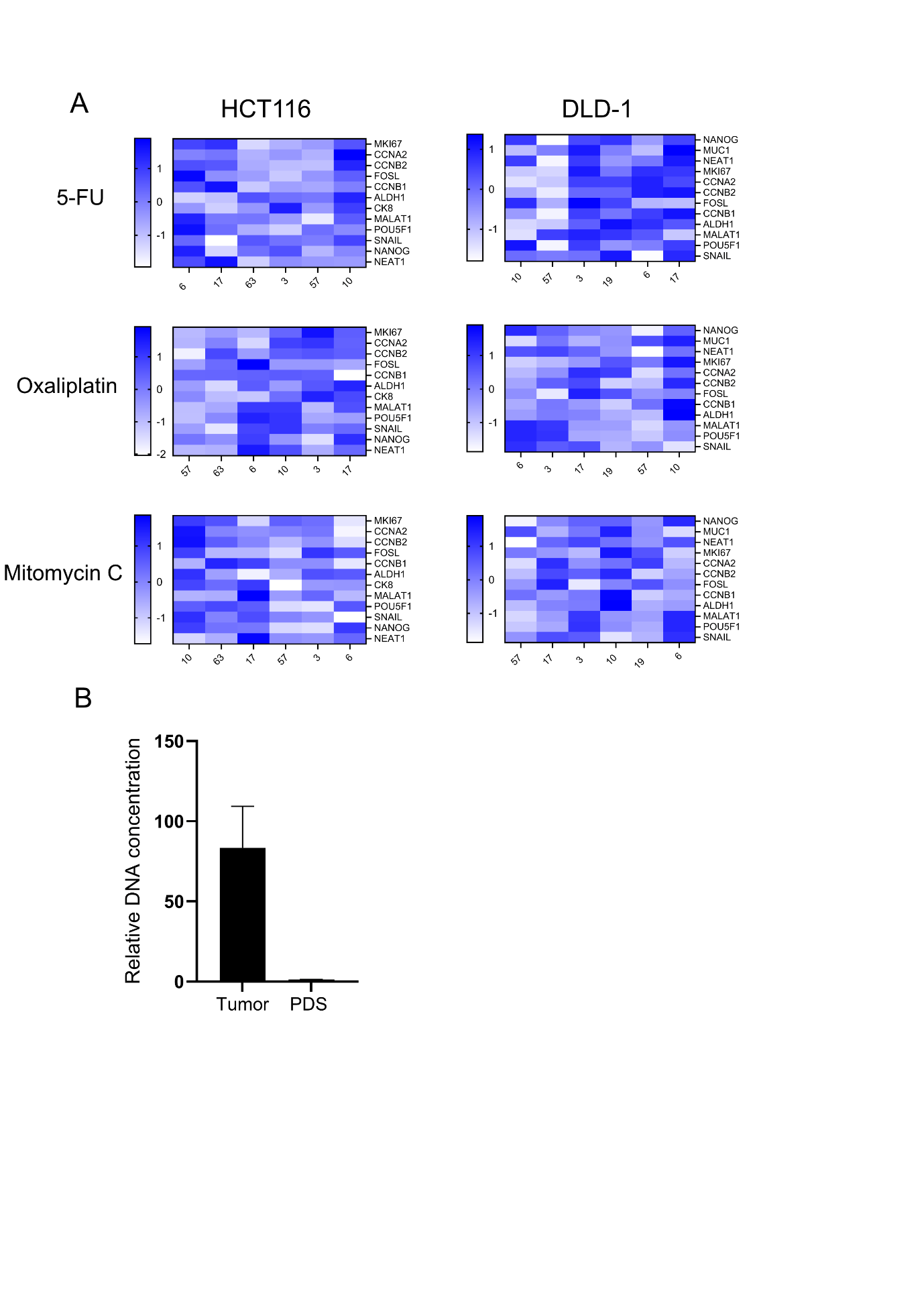


**Supplementary Figure 4: Differential drug responses in a validation cohort of HCT116‑ and DLD‑1–repopulated PDS, and verification of tumor decellularization.**

**(A)** Drug sensitivity was evaluated in a smaller validation cohort consisting of six patient‑derived scaffolds (PDS) repopulated with HCT116 or DLD‑1 colorectal cancer cells. Viability responses to the indicated treatments were quantified and compared between cell line–repopulated PDS in a heat map. **(B)** Successful decellularization of the original tumor tissue prior to scaffold generation was confirmed by DNA quantification using Qubit, demonstrating effective removal of native cellular material before recellularization (n = 3), relative DNA concentration is shown.

# Materials and Methods

Patient material and ethical statement

This study included 75 patients with colorectal adenocarcinomas and peritoneal carcinomatosis. Peritoneal metastases samples were collected at the time of surgery, snap-frozen in liquid nitrogen and stored at -80ºC until use. Informed consent was obtained from all patients, and the study was approved by the regional ethical review board in Gothenburg (DNR 543-17).

Tumor decellularization

Peritoneal metastases samples were decellularized using a protocol adapted from a previous study (9). Briefly, samples were washed once in decellularization buffer containing 0.1% SDS (Sigma-Aldrich), 0.02% Na-Azide (VWR), 5 mM 2H2O-Na2-EDTA (Sigma Aldrich) and 0.4 mM phenylmethylsulfonyl fluoride (Sigma-Aldrich) for 6 hours. Samples were then rinsed in the same buffer without SDS for 15 minutes. Decellularized samples were then washed with distilled water for 72 hours, and subsequently with sterile PBS for 24 hours. All these steps were performed at 37ºC in a 10L Incu-shaker (Benchmark Scientific) with gentle shaking at 175 rpm. PDS were stored at 4ºC in PBS containing 0.02% Na-Azide and 5mM 2H_2_O-Na_2_-EDTA until subsequent use.

Prior recellularization, the samples (now considered PDS) were sterilized in distilled water containing 0.1% paracetic acid (Sigma Aldrich) for 1 hour at room temperature and subsequently in sterile PBS containing 1% Antibiotic-Antimycotic (Thermofisher Scientific) for 24 hours at 37ºC and 175 rpm shaking.

PDS recellularization and drug treatment

HT-29 cells (ATCC HTB-38) and HCT116 (ATCC-CCL-247) were cultured and expanded in McCoy’s 5A modified medium, supplemented with 10% fetal bovine serum, 1% penicillin. DLD-1 (ATCC-CCL221) was cultured in RPMI media supplemented with 10% fetal bovine serum, 1% penicillin/streptomycin and L-glutamin (1 mM) (all ThermoFisher Scientific). Cells were passaged upon reaching 70-80% confluence.

PDS were cut into 1x1x1 mm pieces and pre-conditioned in cell culture medium for 1 hour at 37ºC. Cells were detached from culture plates and seeded onto PDS at a density of 300.000 cells per PDS in 48 well plates. Seeded PDS were incubated at 37ºC for 72 hours. Subsequently and thereafter once a week, PDS were moved to new wells with fresh medium containing 1% Antibiotic-Antimycotic. PDS were cultured for 18 days and subsequently exposed to chemotherapy agents for 48 hours at 37 ºC. Chemotherapy agents used were 5-FU (Teva, 50 mg/mL, Apoteket, Sweden), Mitomycin C (Thermofisher Scientific) and Oxaliplatin (Teva, 5 mg/mL, Apoteket, Sweden).

RNA purification and quantitative PCR

PDS samples were washed in PBS, collected in 350 µL RLT Plus buffer, snap frozen in dry ice until extraction. RNA was purified using RNeasy Plus Micro Kit (Qiagen) and concentration was quantified by NanoDrop (ThermoFisher Scientific).

Complementary DNA synthesis in 20 µL was performed using iScript^TM^ cDNA Synthesis Kit (BioRad) with 100-500 ng of total RNA on a T100 Thermal Cycler (BioRad). The thermal program was: 25ºC for 5 minutes, 46ºC for 20 minutes, 95ºC for 1 minute. The cDNA was diluted 1:5 with RNase free water.

Quantitative polymerase chain reaction (qPCR) was performed on a CFX384 Touch Real-Time PCR Detection System (BioRad) using 6 µL reactions, containing 1X SsoAdvanced Universal SYBR Green Supermix (Biorad), 400 nM of each primer and 2 µL diluted cDNA. The thermal program was: 95ºC for 2 minutes followed by 40 cycles of amplification at 95ºC for 5 seconds, 60ºC for 20 seconds and 70ºC for 20 seconds. All assays were evaluated by melting curve analysis at 65-95ºC with 0.5ºC per 5 seconds increments.

Mutation Analysis

Mutational statuses were available as part of clinical routine in 43 patients, the remaining 32 tumor samples were analyzed as follows. The KRAS G12D (GeneGlobe Cat. No. DMH0000286) and BRAF V600E (GeneGlobe Cat. No. DMH0000004) mutations were analyzed by digital PCR (dPCR) on a QIACuity-One 5-plex System instrument (Qiagen AB, Kista, Sweden). Template DNA (20 ng/reaction) was mixed in QIACuity Nanoplates (26K, Cat. No. 250001) with 10 µL of QIACuity Master Mix (Cat. No. 1133251), 1.3 µL of 30× dPCR LNA Mutation Assay (FAM/HEX, Cat. No. 250200), 1 µL of

Bsu RI restriction enzyme (Thermo Fisher) and RNase-free water to a final volume of 40 µL. The plates were left at room temperature for 10 min before thermal cycling. The dPCR was run on the QIACuity instrument using the following cycling conditions: PCR initial heat activation for 2 min at 95 ◦C, followed by 40 cycles of denaturation for 15 s at 95◦C, and annealing/extension for 30 s at 60◦C. Data analyses were performed using the QIACuity Software Suite version 3.1.1 2024.

Data analysis and statistics

Cycles of quantification (Cq) values were determined by regression with the CFX Manager Software (v3.1, Biorad). Cq values larger than 35 were replaced with the maximum Cq value found in replicates incremented by 1. Missing Cq values were replaced with a value derived from the imputation of other replicates values. Cq values were then normalized to RPS10 and RSP26, (which were confirmed to be the two least variable genes determined by the NormFinder algorithm), transformed to relative quantities through normalization to 2D controls, and finally log2 transformed. Data pre-processing was performed using GenEx software (MultiD). All experiments were conducted by the Minimum Information for Publication of Quantitative Real-Time PCR Experiments guidelines (20).

To calculate the treatment score, gene expression data were first z-score normalized. For each gene category - proliferation, epithelial, epithelial-mesenchymal transition (EMT), and stemness - the expression levels of the associated marker gene were averaged to generate a category score. The overall treatment scores was then computed as a weighted sum of these category scores using the following formula:

Treatment score = - (Proliferation Score + Epithelial Score + EMT Score + Stemness Score). A treatment score was calculated for each treatment and for each patient.

Statistical and survival analysis were performed on GraphPad Prism (v10.4) or SPSS Statistics (v25.0, IBM). Statistical significance of correlations between gene expression values and clinical variables was assessed with Mann-Whitney U test when comparing two groups, or Kruskal-Wallis test when comparing three or more groups. Kaplan–Meier analysis of Progression-free survival, comparing the differences with the log-rank test and with the Gehan-Breslow-Wilcoxon test. Events were defined as relapse or death. Differences were considered significant if p < 0.05. Patients who underwent debulking surgery or open–close procedures were excluded from the Kaplan-Meier analysis. 3 samples were removed due to missing values in the qPCR analysis.

**SUPPLEMENTARY TABLES**

| Gender | Age | PDS from Omental Metastases | Primary Tumor Location | Time of Metastases | PCI | Previous Chemotherapy | Treatment | Completeness of  Cytoreduction | MSS/MSI | KRAS/BRAF |
| --- | --- | --- | --- | --- | --- | --- | --- | --- | --- | --- |
| Female  35 (46,7%) | Min  31 | No  57 (76%) | LS-colon  25 (33,3%) | Synchronous  48 (64%) | PCI<10  21 (28%) | No  28 (37,3%) | CRS + HIPEC  63 (84%) | No visible tumor left  67 (89.3%) | MSI  8 (10,7%) | KRAS  22 (29,3%) |
| Male  40 (53,3%) | Median  63 | Yes  18 (24%) | RS-colon  36 (48%) | Metachronous  27 (36%) | 10<PCI<20  28 (37,3%) | Yes  47 (62,7%) | CRS  5 (6,7%) | <2,5 mm  1 (1,3%) | MSS  57 (76%) | BRAF  18 (24%) |
|  | Max  75 |  | Rectum  4 (5,3%) |  | PCI>20  25 (33,3%) |  | Debulcing  1 (1,3%) | N/A  7 (9,3%) | N/A  10 (13,3%) | WT  35 (46,7%) |
|  |  |  | Appendix  10 (13,3%) |  | N/A  1 (1,3%) |  | Open-Close  6 (8%) |  |  |  |

**Supplementary table 1: Patient’s clinical data.** Abbreviations: LS-colon = Left-sided colon; RS-colon = right-sided colon; PCI = Peritoneal cancer index, CRS = Cytoreductive surgery; HIPEC = hyperthermic intraperitoneal chemotherapy; MSS = Microsatellite stable; MSI = Microsatellite instable.

| Gene marker | Forward sequence (5’-3’) | Reverse sequence (3’-5’) |
| --- | --- | --- |
| Proliferation | | |
| *MKI67* | TGGGTCTGTTATTGATGAGCC | CATCAGGGTCAGAAGAGAAGC |
| *CCNA2* | AAGACGAGACGGGTTGC | GGCTGTTTACTGTTTGCTTTCC |
| *CCNB1* | TTCTGGATAATGGTGAATGGAC | ATGTGGCATACTTGTTCTTGAC |
| *CCNB2* | CGACCCTTGCCACTACACTT | TGACTTCCAATACTTCATTCTCTG |
| Epithelial | | |
| *CK8* | CGACAAGGTAGAGCTGGAGTCT | CGAGCACCACAGATGTGTCCGA |
| *CK18* | CGAGAGACTGGAGCCATTACT | CGAGTCGTGTGATATTGGTGT |
| *MUC1* | CTGGTCTGTGTTCTGGTTGC | CCACTGCTGGGTTTGTGTAA |
| EMT |  |  |
| *SNAIL* | TAATCCAGAGTTTACCTTCCAGCA | AGCCTTTCCCACTGTCCTCA |
| *FOSL1* | GCAGGCGGAGACTGACAA | GGGGAAAGGGAGATACAAGG |
| Stemness | | |
| *ALDH1A3* | AAAAAGAGCGAATAGCACCG | GCATAGAGGGCGTTGTAGCA |
| *MALAT1* | CGACGAGTTGTGCTGCTATC | TCCTCCAAACCCCAAGACCA |
| *NEAT1* | GCCTTCTTGTGCGTTTCTCG | CCCTCCCAGCGTTTAGC |
| *POU5F1* | CTGAGGTGCCTGCCCTTCTA | AGTGTGTCTATCTACTGTGTCCC |
| *NANOG* | CCTATGCCTGTGATTTGTGG | AAGTGGGTTGTTTGCCTTTG |
| *Reference* |  |  |
| *RPS10* | AGCCGCAGAGATGTTGATG | CCTCGGGACTTGAGAGACTG |
| *RPS26* | GATGCGTGCCCAAGGAC | CAGGTCTAAATCGGGGTGG |

**Supplementary table 2: List of primer sequences for qPCR**
